# Supplementary material for: Splice-Junction-Based Mapping of Alternative Isoforms in the Human Proteome
Source: Cell Rep. Author manuscript; Available in PMC 2020 Jan 15. (PMC6961840; doi:10.1016/j.celrep.2019.11.026)
Supplement: 3 [file NIHMS1546469-supplement-3.zip › DF2/PXD009737/TestisTrypsin-220-Q8TEW0-ELKAEDEDIVLTPDGTR.pdf]

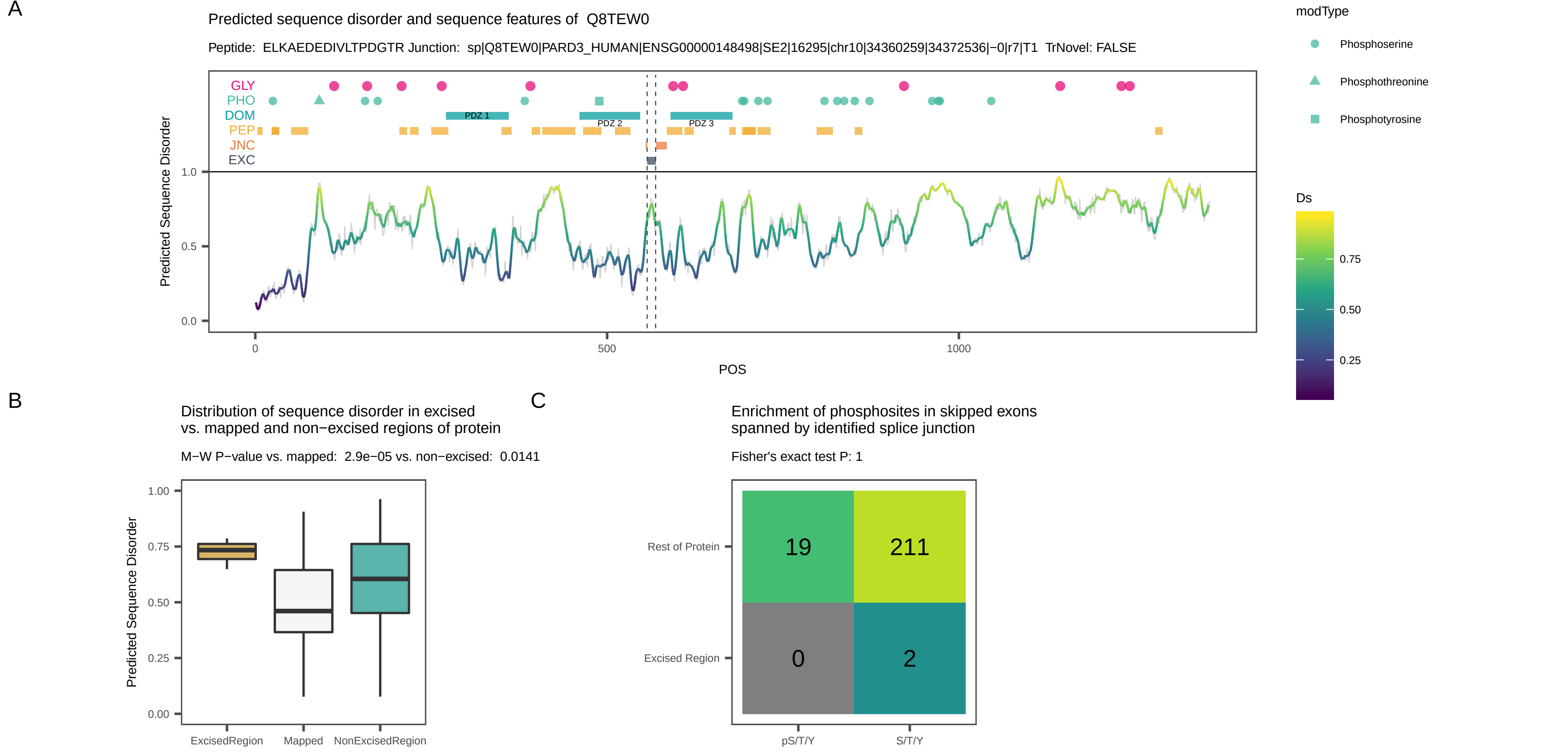
A
Predicted sequence disorder and sequence features of Q8TEW0
Peptide: ELKAEDEDIVLTPDGTR Junction: sp|Q8TEW0|PARD3_HUMAN|ENSG00000148498|SE2|16295|chr10|34360259|34372536|−0|r7|T1 TrNovel: FALSE
GLY
PHO
DOM
PEP
JNC
EXC
PDZ 1
PDZ 2
PDZ 3
Predicted Sequence Disorder
1.0
0.5
0.0
0
500
1000
POS
modType
Phosphoserine
Phosphothreonine
Phosphotyrosine
Ds
0.75
0.50
0.25
B
Distribution of sequence disorder in excised
vs. mapped and non−excised regions of protein
M−W P−value vs. mapped: 2.9e−05 vs. non−excised: 0.0141
Predicted Sequence Disorder
1.00
0.75
0.50
0.25
0.00
ExcisedRegion
Mapped
NonExcisedRegion
C
Enrichment of phosphosites in skipped exons
spanned by identified splice junction
Fisher's exact test P: 1
Rest of Protein
Excised Region
19
211
0
2
pS/T/Y
S/T/Y
